# Skeletal muscle eQTL meta-analysis implicates genes in the genetic architecture of muscular and cardiometabolic traits

**DOI:** 10.1016/j.ajhg.2025.09.003

**Published:** 2025-09-23

**Authors:** Emma P. Wilson, K. Alaine Broadaway, Victoria A. Parsons, Swarooparani Vadlamudi, Narisu Narisu, Sarah M. Brotman, Kevin W. Currin, Heather M. Stringham, Michael R. Erdos, Ryan Welch, Jeffrey K. Holtzman, Timo A. Lakka, Markku Laakso, Jaakko Tuomilehto, Michael Boehnke, Heikki A. Koistinen, Francis S. Collins, Stephen C.J. Parker, Laura J. Scott, Karen L. Mohlke

**Affiliations:** 1 Department of Genetics, University of North Carolina at Chapel Hill, Chapel Hill, NC, USA; 2 Center for Precision Health Research, National Human Genome Research Institute, National Institutes of Health, Bethesda, MD, USA; 3 Department of Biostatistics, Center for Statistical Genetics, University of Michigan, Ann Arbor, MI, USA; 4 Institute of Clinical Medicine, University of Eastern Finland, Kuopio, Finland; 5 Department of Public Health, University of Helsinki, Helsinki, Finland; 6 Department of Public Health and Welfare, Finnish Institute for Health and Welfare, Helsinki, Finland; 7 Research Programs Unit, Clinical and Molecular Metabolism, University of Helsinki, Helsinki, Finland; 8 Department of Medicine, University of Helsinki and Helsinki University Hospital, Helsinki, Finland; 9 Minerva Foundation Institute for Medical Research, Helsinki, Finland; 10 Department of Computational Medicine and Bioinformatics, University of Michigan, Ann Arbor, MI, USA; 11 Department of Human Genetics, University of Michigan, Ann Arbor, MI, USA

**Keywords:** skeletal muscle, eQTL, GWAS, transcriptomics, colocalization, type 2 diabetes, INHBB, signal identification, complex trait, allelic heterogeneity

## Abstract

Identifying genetic variants that regulate gene expression can help uncover mechanisms underlying complex traits. We performed a meta-analysis of skeletal muscle expression quantitative trait locus (eQTL) using data from 1,002 individuals from two studies. A stepwise analysis identified 18,818 conditionally distinct signals for 12,283 genes, and 35% of these genes contained two or more signals. Colocalization of these eQTL signals with 26 muscular and cardiometabolic trait genome-wide association studies (GWASs) identified 2,252 GWAS-eQTL colocalizations that nominated 1,342 candidate genes. Notably, 22% of the GWAS-eQTL colocalizations involved non-primary eQTL signals. Additionally, 37% of the colocalized GWAS-eQTL signals corresponded to the closest protein-coding gene, while 44% were located >50 kb from the transcription start site of the nominated gene. To assess tissue specificity for a heterogeneous trait, we compared colocalizations with type 2 diabetes (T2D) signals across muscle, adipose, liver, and islet eQTLs; we identified 551 candidate genes for 309 T2D signals representing 36% of T2D signals tested and over 100 more than were detected with any one tissue alone. We then functionally validated the allelic regulatory effect of an eQTL variant for *INHBB* linked to T2D in both muscle and adipose tissue. Together, these results further demonstrate the value of skeletal muscle eQTLs in elucidating mechanisms underlying complex traits.

## Teaser

To investigate the underlying mechanisms of complex traits, we performed an eQTL meta-analysis in skeletal muscle and identified 18,818 conditionally distinct signals for 12,283 genes. Colocalization with GWASs nominated 1,342 genes with potential disease relevance, and integration across eQTL tissues nominated genes for 36% of type 2 diabetes GWAS signals.

## Introduction

Genome-wide association studies (GWASs) have identified thousands of genomic regions associated with cardiometabolic diseases and related traits.^1^^,^^2^ However, over 90% of GWAS signals are in non-coding regions, suggesting that most causal variants exert their effects through gene regulation.^3^^,^^4^^,^^5^^,^^6^ To uncover the mechanisms underlying these non-coding signals, GWAS data can be integrated with expression quantitative trait loci (eQTLs), which link genetic variants with changes in gene expression. Colocalization of GWAS and eQTL signals can identify variants that may influence both a trait and gene-expression levels and therefore prioritize candidate genes.^5^^,^^7^^,^^8^^,^^9^ Multiple variants can independently influence expression of a single gene, and identifying these distinct association signals can reveal additional regulatory mechanisms.^10^^,^^11^^,^^12^^,^^13^^,^^14^^,^^15^

A popular method to identify conditionally distinct signals in meta-analyses is through approximate conditional analysis of summary statistics, as implemented in GCTA-COJO^16^ and SuSiE.^17^ While these methods are computationally efficient and flexible, their accuracy depends on the availability of a large reference panel that closely matches the population structure of the meta-analysis participants, and the resulting signal identification remains inherently approximate. In contrast, All-in-one Package for Efficient Xqtl analysis (APEX)^18^ uses individual-level data to form study-specific variance-covariance matrices. This strategy allows for efficient and accurate conditional analysis that improves detection of non-primary eQTL signals and robust GWAS colocalizations, providing deeper understanding of trait genetic architecture.^11^^,^^12^

Skeletal muscle is a particularly compelling tissue for understanding cardiometabolic traits, as it functions as both an effector and responder to metabolic stress.^19^^,^^20^^,^^21^ Skeletal muscle plays a central role in cardiometabolic health by regulating glucose uptake and lipid utilization.^22^^,^^23^^,^^24^ Disruption of these processes in muscle tissue contributes to dyslipidemia, obesity, diabetes, and cardiovascular disease.^19^^,^^20^ Skeletal muscle’s response to insulin is the primary determinant of insulin sensitivity and peripheral insulin resistance.^25^ Given its role in both energy storage and expenditure, genetic variants that influence gene expression in muscle may exert broad effects on cardiometabolic traits.^26^^,^^27^ Identifying eQTLs in muscle could thus help identify genes critical to the pathogenesis of cardiometabolic diseases.

For complex traits, such as type 2 diabetes (T2D), multiple tissues can contribute to disease risk.^28^^,^^29^^,^^30^^,^^31^ In such cases, integrating GWASs with eQTLs from multiple disease-relevant tissues can improve gene-trait mapping. Although T2D can be characterized by insufficient insulin production from the pancreatic islet beta cells, increased peripheral insulin demand—required for adequate glucose uptake—is also a critical component.^24^^,^^28^ Skeletal muscle is a major site of insulin-stimulated glucose disposal, but insulin regulation and glucose uptake also occur in adipose tissue and liver. Identifying eQTLs across these key tissues may help pinpoint candidate genes involved in T2D pathogenesis and enhance our understanding of tissue-specific genetic regulation.

Here, we present a skeletal muscle eQTL meta-analysis of 1,002 individuals from two studies. We identified conditionally distinct muscle eQTL signals and colocalized them with GWAS signals from 26 disease-relevant traits. We further performed colocalization analyses with eQTLs from four T2D-relevant tissues and T2D GWAS signals. At one T2D signal that colocalized with eQTLs for the same gene in both muscle and subcutaneous adipose tissues, we functionally validated the allelic regulatory effect. Through these analyses, we expanded our understanding of muscle eQTL architecture and highlighted candidate genes and mechanisms that may influence cardiometabolic traits.

## Methods

### Ethics declaration

The Finland-United States Investigation of Non-Insulin Dependent Diabetes Mellitus (FUSION) study was approved by the Coordinating Ethics Committee of the Hospital District of Helsinki and Uusimaa, and written informed consent was obtained from all participants.

### Study cohorts

#### Genotype-Tissue Expression Consortium

The generation and processing of samples, whole-genome sequencing (WGS), RNA sequencing (RNA-seq), and quality control in the Genotype-Tissue Expression (GTEx) Consortium (v.8) have been described previously.^7^ We obtained WGS genotype files from dbGaP (dbGaP: phs000424). We downloaded gene-level read counts per individual from the GTEx portal and analyzed the 706 skeletal muscle samples for which both expression and genotype data were available.^7^

#### FUSION

Sample preparation and quality control for the FUSION tissue collection have been described previously.^32^^,^^33^ We obtained WGS fastq files for 326 FUSION study participants from dbGaP (dbGaP: phs001048.v3.p1, phs001579.v1.p1) and aligned paired-end sequence reads to the hg38 reference genome,^34^ performed quality control checks using SAMTOOLS (v.1.9),^35^ and validated WGS data against 2.5M exome chip data^32^ using VerifyBamID (v.1.1.1).^36^ We assessed family relationships with KING (v.1.4)^37^ and only analyzed one member of each pair of first- or second-degree relatives. We retained reads with mapping quality ≥30 and called germline single nucleotide polymorphisms (SNPs) and indels following recommendations of GATK best practices.^38^ We computed the genotype likelihood of each sample at a given site, assigned by the GATK “HaplotypeCaller” function. Final SNP and indel genotypes of the samples were assigned jointly by the “GenotypeGVCFs” function. Variant recalibration models were constructed separately for SNPs and indels using “VariantRecalibrator.” For SNPs, training resources included 1000G phase 1, HapMap, Omni2.5 chip, and dbSNP 151 SNPs. For indels, we used the Affymetrix Axiom Exome chip, dbSNP 151 SNPs, and 1000G known indels. We then applied the models to the variants with “ApplyVQSR” and assigned genotype quality scores.

We obtained skeletal muscle RNA-seq fastq files,^32^^,^^33^ realigned the reads to the hg38 reference genome, quantified expression levels using gene annotations from GENCODE v.19, and generated a raw read count matrix of genes by sample, as previously described.^39^ We analyzed 296 samples for which genotype data were available.

### Study-level eQTL analyses

While both GTEx and FUSION have previously reported eQTLs, APEX uses individual-level data to model study-level variance-covariance matrices prior to meta-analysis and detection of multiple signals. Within each study, we removed lowly expressed genes (<5 counts in ≥25% of the individuals) and calculated counts per million (CPM), which we normalized using the trimmed mean of M values (TMM) in edgeR.^40^^,^^41^ We then inverse-normal transformed the counts and annotated genes using the hg38 GENCODE v.26 gene transfer format (GTF) file as modified by GTEx (see web resources).^7^ We used this file to annotate the study-provided ENSEMBL IDs with gene names, transcription start site (TSS) locations, and gene biotype. We retained autosomal, bi-allelic variants with minor allele frequency (MAF) ≥0.01 within 1 Mb of the gene’s TSS, including all genes expressed in either study. For the multi-population GTEx samples, we incorporated three genotype principal components as described previously.^7^

To account for technical variation, we calculated PEER factors (v.1.3)^42^ using the inverse-normal transformed gene-expression residuals as described previously.^12^ We tested sets of PEER factors ranging from 0 to 100 by 10 to include as covariates for each study. We used a significance threshold of *p* ≤ 1 × 10^−5^ and defined an eGene as a gene for which at least one variant was significantly associated with gene expression. For each set of PEER factors, we performed eQTL analysis using APEX^18^ and counted the number of eGenes. We chose the final number of PEER factors as the number after which the increase in eGenes was <1%: 40 for GTEx and 20 for FUSION.

### Meta-analyses

We performed a meta-analysis of the GTEx and FUSION eQTLs using APEX^18^ with a significance threshold of *p* ≤ 1 × 10^−5^ and including all genes tested in either study. We included data in the results tables to indicate which studies contributed to a given gene, called “Studies (1 = FUSION, 2 = GTEx),” with “1,2” indicating that the gene was present in both studies. We then used APEX to identify distinct association signals through fixed-effect stepwise conditional analyses. To enable direct comparison across studies at the same significance threshold, we also performed conditional analyses using the study-level summary statistics. For comparison, we calculated false discovery rate (FDR) as described previously^11^^,^^12^^,^^43^ and confirmed that *p* ≤ 1 × 10^−5^ is more stringent than FDR 5%.

To compare the signals identified in GTEx, FUSION, and the meta-analysis, we applied the same APEX pipeline and *p* ≤ 1 × 10^−5^ threshold to identify eGenes and association signals in each study. To compare the primary eQTL meta-analysis results to the skeletal muscle eGenes reported in GTEx v.8,^7^ we required that eGenes meet the significance thresholds established by both studies. Our significance threshold (*p* ≤ 1 × 10^−5^) was more stringent than the threshold used in GTEx (FDR ≤ 5%).

### eQTL signal isolation

To test eQTL signals for colocalization with GWAS signals and meet software assumption of only one signal in each region,^9^ we “isolated” each eQTL signal from all other eQTL signals for the same gene. As described previously,^11^^,^^12^ we used Apex2R^18^ (see web resources) on each eGene that contained two or more signals; we conditioned on all other signals for that eGene and generated the conditional summary statistics for the target signal as the isolated signal summary statistics. For eGenes that contained exactly one signal, we used the APEX single-variant meta-analysis summary statistics, termed “marginal” summary statistics.

### eQTL signal characterization

We numbered signals for each eGene using APEX’s forward-reverse selection. Starting with the most significant marginal variant, we iteratively added the top conditional variant and, after each addition, removed any variants no longer significant when conditioned on the others. This process continued until no further variants could be added or removed, yielding a final set of mutually significant signals, which we numbered in the order they were identified. We used all 1,339 eGenes that contained three or more distinct signals to compare characteristics of the primary, secondary, and subsequent signals, termed “tertiary+.” We used the absolute value of each isolated signal’s conditional *β* value to compare effect sizes. As APEX did not output pooled effect allele frequencies for each variant in the meta-analysis, we ran a parallel meta-analysis using METAL^44^ to obtain the pooled allele frequencies and appended them to the APEX output for downstream analyses. We used GENCODE v.26 to calculate the absolute distance from each signal’s lead variant to the gene’s TSS. We used Mood’s median test to evaluate the differences between each signal number’s distance to the TSS, magnitude of effect size, and MAF.

### GWAS data for colocalization

We downloaded GWAS summary statistics for 26 muscular and cardiometabolic traits.^45^^,^^46^^,^^47^^,^^48^^,^^49^^,^^50^^,^^51^^,^^52^^,^^53^^,^^54^^,^^55^^,^^56^^,^^57^ Dataset access is provided in the supplemental information. We isolated conditionally distinct GWAS signals within each trait using GCTA cojo-slct and cojo-cond functions^16^^,^^58^ with a reference panel of 40,000 unrelated European-ancestry individuals in the UK Biobank (UKB)^54^ as described previously.^11^^,^^12^ As our meta-analysis included approximately 90% European-ancestry individuals, we used European-ancestry subsets of GWASs, when available, to better align populations for linkage disequilibrium (LD) comparisons.

### Colocalization with GWAS signals

To compare eQTLs to hg19 GWAS data, we lifted over the eQTL meta-analysis summary results from hg38 to hg19 using the variant look-up file provided by GTEx (see web resources).^7^

As an initial filtering step, we used PLINK (v.1.90b3)^59^ to calculate the LD *r*^2^ between all muscle eQTL lead variants and all GWAS lead variants within 500 kb for each trait using the EUR UKB LD reference panel. We next ran coloc (v.5.1.0.1)^9^ on all eQTL-GWAS signal pairs that showed at least moderate LD between their lead variants (*r*^2^ ≥ 0.5). We included all variants present in both sets of summary statistics and located within 500 kb of the GWAS lead variant. coloc calculates the posterior probability that the tested GWAS and eQTL regions either have no association signals (PP_H0_), have a signal in only one dataset (PP_H1_ or PP_H2_), both have a signal that are driven by different causal variants (PP_H3_), or both have a signal that share the same causal variant, i.e., are colocalized (PP_H4_). We used a coloc PP_H4_ threshold ≥0.7 to consider an eQTL-GWAS signal pair colocalized, and report the sensitivity of that pair’s PP_H4_ outcome to varying the prior parameters.

To characterize skeletal muscle cell-type expression of colocalized genes, we report mean CPM from single-nucleus RNA-seq data per cell type across 281 individuals.^60^

### T2D colocalizations across tissues

To compare T2D colocalizations from Suzuki et al.^45^ with eQTLs from four tissues, we used publicly available eQTL data for subcutaneous adipose, liver, and pancreatic islets.^11^^,^^12^^,^^61^ The adipose and liver datasets were analyzed using a similar pipeline in APEX and included conditionally isolated signals. For islets, as this publicly available eQTL dataset was analyzed using fastQTL and did not include isolated signals, we had to assume only one signal per gene. We tested for colocalization between isolated T2D signals and each of the eQTL datasets using the pipeline as described above.

Among the 44 genes colocalized with T2D in both adipose and muscle, we prioritized *INHBB* for functional follow-up based on several factors: the presence of regulatory annotations suggesting the region harbors an active regulatory element, the absence of high-LD proxies with *INHBB* lead variant rs11688682, and the gene’s role in cardiometabolic traits in adipose and muscle.^62^^,^^63^^,^^64^

### Cell culture

We maintained all cultures at 37°C in a humidified incubator with 5% CO_2_. Simpson-Golabi-Behmel syndrome (SGBS) human preadipocytes^65^ were generously provided by Dr. Martin Wabitsch (University of Ulm) and cultured in supplemented DMEM/F12 (Corning) medium supplemented with fetal bovine serum (FBS) (10%), biotin (33 μM), and pantothenate (17 μM). SGBS preadipocytes were differentiated for 4 days by culturing in serum-free basal medium containing transferrin (10 μg/mL), insulin (20 nM), cortisol (200 nM), triiodothyronine (T3) (400 pM), dexamethasone (50 nM), IBMX (500 μM), and rosiglitazone (2 μM). We cultured LHCN-M2 human myoblasts^66^ (Evercyte, Vienna, Austria) in DMEM-high glucose/medium 199 (ratio 4:1) (Gibco) supplemented with FBS (15%), HEPES (0.02 M), zinc sulfate (0.03 μg/mL), vitamin B12 (1.4 μg/mL), dexamethasone (0.055 μg/mL), recombinant human hepatocyte growth factor (2.5 ng/mL) (Pepro Tech, 100-39), and human fibroblast growth factor-basic (FGF-2/bFGF) (10 ng/mL) (Pepro Tech, 100-18B). To differentiate myoblasts into myocytes, we cultured cells in DMEM containing glucose (5.5 mM) and horse serum (2%), with media changes every day for 5 days.

### Transcriptional reporter assays

We performed luciferase transcriptional reporter assays to test rs11688682 for allelic differences in transcriptional activity. We amplified a 610 bp region that spans a chromatin accessibility peak,^67^ includes histone marks suggestive of enhancer activity,^68^ and contains rs11688682 from individuals homozygous for each allele using PCR with primers containing sequence complementary to the genome (5′-TCTGGGCTCTTTCCAGTTTG-3′ and 5′-TCCTCATGGGTCAAGATGGT-3′) and added KpnI and XhoI restriction sites. As described previously,^67^ amplicons were cloned into the Firefly luciferase reporter vector pGL4.23 (Promega) in both orientations with respect to the genome. Five independent clones per construct were isolated and verified by Sanger sequencing. We tested transcriptional activity in preadipocytes, differentiated adipocytes, myoblasts, and differentiated myocytes. Cells were cotransfected in triplicate using each of five replicate plasmid clones or a pGL4.23 negative control with phRL-TK Renilla luciferase reporter vector (Promega) using Lipofectamine 3000. We measured luciferase activity using the Dual-Luciferase Reporter Assay System (Promega) 28 h after transfection for preadipocytes and adipocytes and 48 h after transfection for myoblasts. To measure luciferase activity in myocytes, we transfected LHCN-M2 myoblasts and cultured them for 24 h before differentiation for 5 additional days before assaying. We first normalized Firefly luciferase activity to Renilla activity and then normalized to the average of two negative controls (relative luciferase activity). For each of the five replicate plasmid clones, we averaged the triplicate values. All experiments were carried out on a second day and showed equivalent results. We tested for differences in relative Firefly luciferase activity between alleles using two-sided t tests.

We determined the expression of *INHBB* in undifferentiated SGBS cells and SGBS cells differentiated for 4 days using previously published RNA-seq data.^67^ We aligned SGBS RNA-seq reads and quantified gene expression as described previously.^67^ We used tximport^69^ to calculate length-scaled transcripts per million (TPM) and used the median TPM across replicates to measure gene expression. We used ENCODE RNA-seq data^68^^,^^70^ to determine the expression of *INHBB* in undifferentiated LHCN-M2 cells (ENCODE accession ENCFF369ZZI) and LHCN-M2 cells differentiated for seven days (ENCODE accession ENCFF314BVO); gene expression was measured using TPM.

## Results

### Skeletal muscle eQTL meta-analysis

**Figure 1:**
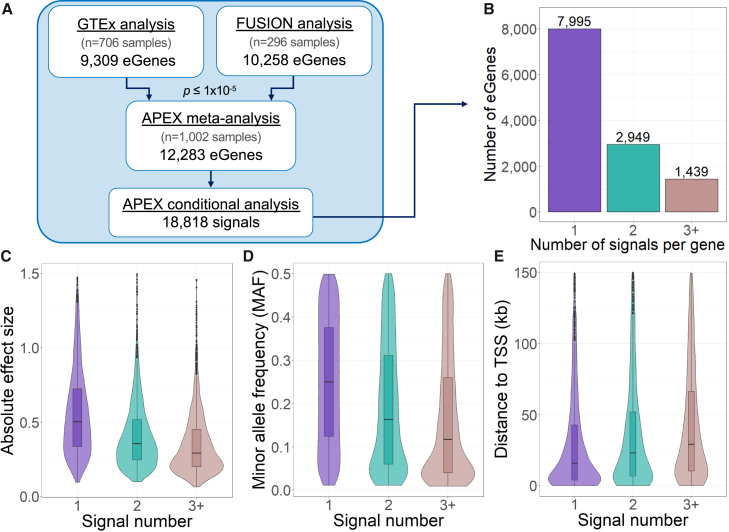
Skeletal muscle eQTL gene and signal characteristics (A) Study design for eQTL discovery. eQTL signals were identified based on a threshold of *p ≤* 1 × 10^-5^. (B) Number of signals identified per gene. (C–E) Characteristics of the primary, secondary, and tertiary+ signals for 1,339 eGenes that contained three or more eQTL signals. (C) absolute effect size, (D) minor allele frequency (MAF), and (E) distance to the gene’s transcription start site (TSS).

We conducted a skeletal muscle eQTL meta-analysis combining the FUSION and GTEx studies, with a total sample size of 1,002 individuals. We analyzed 24,071 genes and 7.95 million autosomal variants (MAF ≥ 0.01) (Tables 1 and S1; Figure 1A). We identified 18,818 conditionally distinct signals across 12,283 eGenes at a significance threshold of *p* ≤ 1 × 10^−5^ (Figure 1B; Tables S2 and S3). Using the same analysis pipeline and significance threshold, the meta-analysis yielded 20%–32% more eGenes and 28%–35% more eQTL signals than either study alone (Table 1, Figure S1, and methods). Of the 12,283 eGenes identified in the meta-analysis, 30% were not detected as significant in GTEx alone, 27% were not detected in FUSION alone, and 9% were not detected in either study alone (Figure S2). Of the 12,283 eGenes identified in the meta-analysis (*p* ≤ 1 × 10^−5^), almost all (12,281) also passed the GTEx FDR ≤ 5% threshold (Tables S2 and S3). In contrast, GTEx only identified 10,545 eGenes (of 13,165 reported) that also met our stricter *p* value threshold (Figure S3). The larger meta-analysis and rigorous significance threshold had an enhanced ability to detect eQTLs.

**Table 1:** Skeletal muscle eQTL signals detected in individual studies and by meta-analysis

**eQTL analysis**				**Number of eGenes (% of eGenes) with the indicated number of signals**					
Study	Genes analyzed	Significant eGenes	Distinct signals	1	2	3	4	5	6+
FUSION (*n* = 296)	21,866	10,258	14,660	7,184 (70%)	2,277 (22%)	525 (5.1%)	148 (1.4%)	65 (0.6%)	59 (0.6%)
GTEx (*n* = 706)	21,420	9,309	13,957	6,343 (68%)	2,052 (22%)	537 (5.8%)	202 (2.2%)	86 (0.9%)	89 (1.0%)
Meta-analysis (*n* = 1,002)	24,071	12,283	18,818	7,995 (65%)	2,949 (24%)	865 (7.0%)	273 (2.2%)	100 (0.8%)	101 (0.8%)

Number of genes with from one to six or more eQTL signals (*p* ≤ 1 × 10^−5^), identified in each study and the meta-analysis. The percentage of eGenes with that number of observed signals is shown in parentheses.

**Figure 2:**
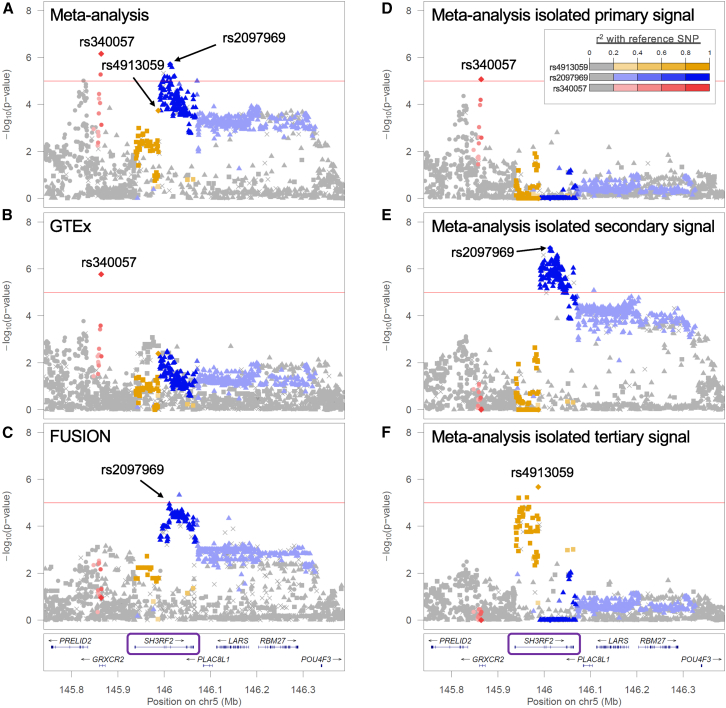
Three skeletal muscle eQTL signals for *SH3RF2* identified in the meta-analysis (A–C) *SH3RF2* was identified as an eGene with (A) three conditionally distinct signals at rs340057, rs2097969, and rs4913059 in the meta-analysis, (B) one signal at rs340057 in GTEx, and (C) one signal at rs72818497 (*r*^2^ = 0.48 with rs2097969) in FUSION. (D–F) Each of the three signals identified in the meta-analysis was isolated by conditioning on the other two signals. All plots are colored by linkage disequilibrium (LD) with the lead variants of the three signals as identified in the meta-analysis. Red lines indicate the significance threshold (*p* ≤ 1 × 10^−5^).

In the meta-analysis, 35% of the eGenes contained two or more distinct signals, a higher proportion than we found in either single-study analysis (Table 1). For example, three distinct eQTL signals were identified for *SH3RF2* in the meta-analysis (lead variants rs340057, rs2097969, and rs4913059), while each contributing study identified only one signal. The primary meta-analysis signal was the only signal detected in GTEx (rs340057), while a variant in moderate LD with the meta-analysis secondary signal was the only signal identified in FUSION (rs72818497: *r*^2^ = 0.48 with rs2097969) (Figure 2). All three meta-analysis signals were driven by common variants (meta-analysis MAF >0.10; Table S4). This example illustrates how the increased power of the meta-analysis enabled detection of multiple regulatory signals not identified in the individual studies. The three distinct signals associated with this eGene may represent separate causal variants that independently affect *SH3RF2* expression in muscle tissue.

To better understand the characteristics of skeletal muscle eQTL signals that may influence the order in which they were discovered in the stepwise conditional analysis, we compared the signals detected as first, second, or subsequently in rank, termed primary, secondary, and tertiary+ signals, respectively. Of the 1,339 eGenes with three or more conditionally distinct eQTL signals, primary signals were typically closer to the gene TSS (median distance of 20 kb for primary vs. 66 kb for tertiary+, Mood’s *p* value = 4 × 10^−51^), had larger absolute effect sizes (median absolute *β* 0.51 vs. 0.29, *p* = 2 × 10^−88^), and comprised more common variants (median MAF = 0.25 vs. 0.12, *p* = 3 × 10^−53^). These patterns are consistent with findings from previous studies of multi-signal eGenes in other tissues^10^^,^^11^^,^^12^ (Figures 1C–1E).

### Skeletal muscle eQTL colocalization with GWAS signals

We used the muscle eQTL meta-analysis results to identify candidate genes through colocalization with GWAS signals for 26 muscular or cardiometabolic traits (Table S5), analyzing conditionally isolated skeletal muscle eQTL signals and isolated GWAS signals from each study (see methods). In total, we identified 2,252 colocalizations for 1,342 eGenes (Tables 2, S6, and S7). We characterized skeletal muscle cell-type expression of the colocalized eGenes and found that 60%–75% were at least nominally expressed (mean CPM ≥1) within each cell type (Table S8).^60^ Of the 1,342 eGenes that had at least one GWAS-eQTL colocalization, 24% were not detected as eGenes in GTEx, 24% were not detected in FUSION, and 8% were not detected in either study individually. We next investigated non-primary eQTL signals and found that 22% of eQTL signals that colocalized with GWAS signals were not the first eQTL signal detected for a gene. These results demonstrate that weaker eQTL signals only detectable in the meta-analysis can still play a critical role in prioritizing candidate genes for GWAS signals.

**Table 2:** Skeletal muscle eQTL colocalizations with GWAS traits

**Study**	**GWAS signals included in analysis**	**Colocalizations (PP_H4_ ≥ 0.7)**	**eGenes with ≥1 colocalized eQTL**
Appendicular lean mass	1,035	185	184
Type 2 diabetes^∗^	862	179	179
Body mass index	941	151	150
Triglycerides (log)^∗^	705	145	142
High-density lipoprotein chol^∗^	816	132	131
Total chol^∗^	828	128	127
Creatinine^∗^	541	119	117
Non-high-density lipoprotein chol^∗^	604	112	112
White blood cell	637	111	110
Hip circumference^∗^	471	102	101
Diastolic blood pressure	402	100	93
Low-density lipoprotein chol^∗^	687	96	96
Waist circumference^∗^	395	85	85
C-reactive protein^∗^	438	84	84
Alkaline phosphatase	289	78	75
Waist-to-hip ratio adjBMI	463	75	75
Waist-to-hip ratio	382	68	68
Systolic blood pressure	339	65	65
Pulse pressure	329	64	64
Coronary artery disease	241	54	53
Alanine transaminase	150	33	33
Gamma-glutamyltransferase	200	29	29
Hemoglobin A1c^∗^	102	25	24
Vitamin D	143	19	19
Fasting glucose	99	9	9
Fasting insulin	44	4	4

Summary of colocalization results across 26 GWAS traits. Study names with an asterisk (^∗^) denote studies for which we ran GCTA to identify conditionally distinct signals. For studies that provided both multi-population and European-stratified summary statistics, we used the European subset. Columns show the number of colocalized GWAS-eQTL signal pairs and the number of eGenes that have at least one colocalized eQTL signal with that trait (coloc posterior probability PP_H4_ ≥ 0.7). Across all traits, GWAS signals colocalized with eQTL signals for 1,342 unique eGenes. chol, cholesterol; adjBMI, adjusted for body mass index.

**Figure 3:**
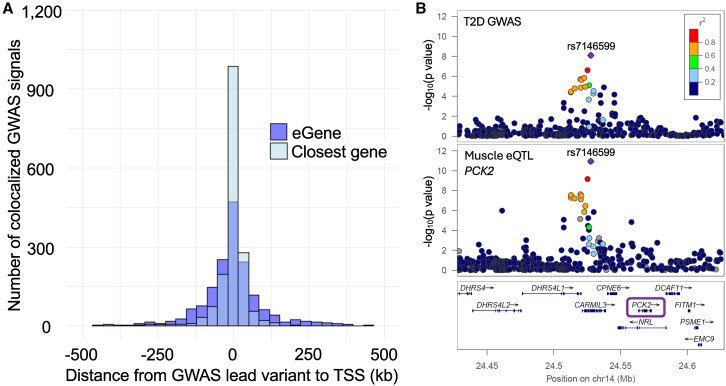
GWAS signals did not always colocalize with the nearest protein-coding gene (A) For the 1,713 colocalizations with an eQTL for a protein-coding gene, bars show the distance from the GWAS lead variants to the TSS of the nearest protein-coding gene and the colocalized eGene. Plot truncated at ±500 kb. (B) Example T2D GWAS signal colocalized with a muscle eQTL for the third-closest protein-coding gene, *PCK2*.

Although GWAS signals are sometimes assigned to the nearest gene as a proxy for identifying candidate genes, our colocalization analyses suggest that this approach can overlook genes that are more likely to be functionally linked to the signal. Most GWAS leads are near their colocalized eGene: 56% of the GWAS leads were located within 50 kb of the TSS of their colocalized eGene, 76% within 100 kb, and 92% within 250 kb. However, among colocalizations involving a protein-coding eGene, only 37% corresponded to the closest protein-coding gene (Figures 3A and S4; Table S7). For example, a T2D signal with lead variant rs7146599 has 14 nearby genes within 100 kb; it is located in an intron of *CARMIL3* and lies only 12 kb from *CPNE6*. However, this T2D signal colocalized exclusively with a muscle eQTL for *PCK2* (PP_H4_ = 1, same lead variant) (Figure 3B). *PCK2* is the fourth closest gene, located 36 kb from the variant, and the T2D risk allele is associated with increased *PCK2* expression. These results underscore that physical proximity alone is insufficient to identify likely causal genes at a GWAS signal.

### T2D signal colocalization with eQTLs across tissues

**Figure 4:**
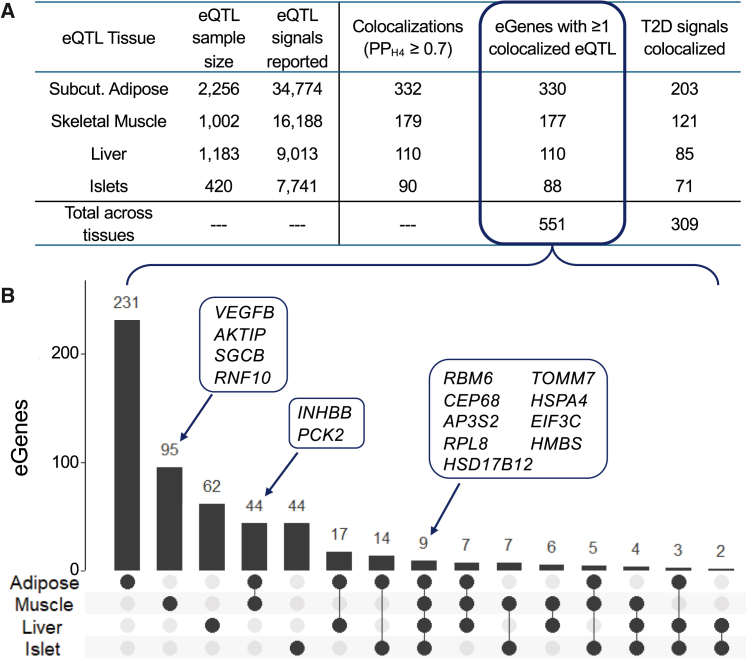
Colocalizations between T2D and eQTLs across four tissues The 862 T2D signals we identified in the GGI-European analysis (Suzuki et al.^45^) were tested for colocalization with eQTLs from four tissues. (A) Summary of the total number of colocalized (posterior probability PP_H4_ ≥ 0.7) GWAS-eQTL signal pairs and the numbers of eGenes and T2D signals with at least one colocalized eQTL signal. The “Total across tissues” row shows the number of unique eGenes/T2D signals with at least one colocalization in at least one tissue. (B) UpSet plot showing eGenes linked to T2D signals across tissues. For each gene that had an eQTL signal colocalized with at least one T2D signal in at least one tissue, bars show which tissue(s) identified a colocalization with that gene. Many of the eGenes that only show evidence of colocalization in one tissue may not have been detected as eQTLs in the other tissues due to limited eQTL discovery power, low gene-expression levels, or differences in data sources or quality.

T2D is driven by distinct mechanisms that act across different tissues. To identify additional candidate genes for T2D GWAS signals and to better understand how gene regulation may act across various tissues, we focused on four tissues relevant to T2D pathophysiology: skeletal muscle, subcutaneous adipose, liver, and pancreatic islets. Of 862 tested T2D signals,^45^ we colocalized 309 (36%) with an eQTL in at least one tissue, identifying 551 candidate target genes (Figure 4A).^11^^,^^12^^,^^61^ Testing across all four tissues enabled us to colocalize over 50% more T2D signals than using any single tissue alone (Figure 4A and Table S9). In skeletal muscle, 121 T2D signals colocalized with eQTL signals for 177 genes, of which 95 genes were uniquely identified in muscle (Figure 4B), including *VEGFB*, *AKTIP*, *SGCB*, and *RNF10*. The four eQTL datasets vary greatly in sample size and power to detect eQTLs (Figure S5) and may differ in gene-expression data quality and characteristics. The highest number of T2D colocalizations was observed in adipose tissue (203 signals), which was expected given the adipose eQTL study’s approximately 2-fold larger sample size compared with the other tissues and its greater power to detect eQTL signals. In pancreatic islets, we identified 71 T2D signals that colocalized with eQTL signals for 88 genes, despite the eQTL study’s relatively small sample size and its analysis being limited to primary signals only. While some colocalizations appeared unique to a single tissue, these may be detected in future eQTL studies with increased sample size and resolution.

We identified nine genes as candidates for T2D based on eQTL colocalization across all four tissues (Figure 4B). Six of these genes—*RBM6*, *CEP68*, *AP3S2*, *RPL8*, *TOMM7*, and *HSD17B12—*have previously been implicated in T2D risk,^71^^,^^72^^,^^73^^,^^74^ while the remaining three—*HSPA4*, *EIF3C*, and *HMBS—*are broadly expressed housekeeping genes. For these colocalizations, the T2D risk alleles were associated with lower expression of *RBM6*, *RPL8*, and *HSD17B12* and with higher expression of the other six genes, with consistent effect direction across all four tissues (Figure S6). Some of these shared eQTLs also colocalized with other cardiometabolic traits in muscle. For example, the muscle eQTL for *HSPA4*, which encodes a heat-shock protein, also colocalized with GWAS signals for high-density lipoprotein cholesterol, triglycerides, alkaline phosphatase, and diastolic blood pressure (Figure S7). Similarly, the muscle eQTL for *HMBS*, which encodes hydroxymethylbilane synthase, also colocalized with GWAS signals for body mass index, waist-hip ratio, and waist circumference (Figure S8). These cross-trait colocalizations may indicate shared regulatory mechanisms underlying T2D and related cardiometabolic phenotypes. Overall, these nine genes are supported by multiple lines of evidence and represent strong candidates for involvement in T2D pathogenesis.

### Functional validation of *INHBB* locus lead variant

**Figure 5:**
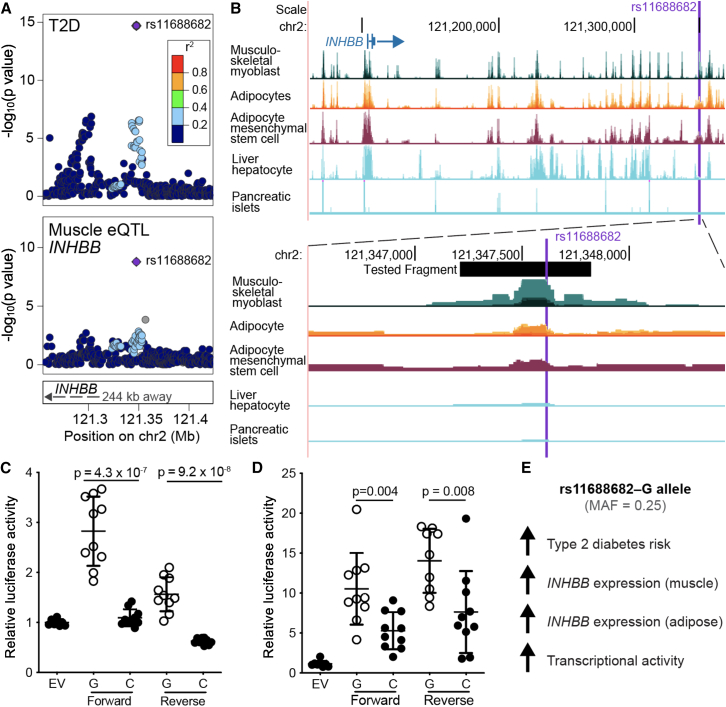
Allelic effects of rs11688682 in putative regulatory elements in myoblasts and adipocytes (A) Colocalized T2D GWAS (top) and skeletal muscle eQTL for *INHBB* (bottom), both with lead variant rs11688682. (B) rs11688682 is located 244 kb downstream of *INHBB* in a region of accessible chromatin (ATAC-seq) in at least myoblasts and adipocytes. (C and D) Transcriptional reporter assay results of a 610 bp fragment surrounding rs11688682, which showed enhancer activity in (C) LHCN-M2 myoblasts and (D) SGBS-derived differentiated adipocytes. EV, empty vector. The 7–10 points per allele or EV represent independent transfections and show the average of triplicate luciferase values. Bars show standard deviations; *p* values correspond to two-sided t tests. (E) Cartoon summarizing the directions of effect of the rs11688682^G^ allele associations with T2D risk and *INHBB* expression, and the observed effect on transcriptional activity.

We further investigated one GWAS signal that colocalized with an eQTL for the same gene, *INHBB*, in both skeletal muscle and subcutaneous adipose tissue and that harbored a strong candidate regulatory variant. A T2D signal on chromosome 2 with lead variant rs11688682 colocalized with the primary eQTL signal for *INHBB* in both tissues (PP_H4_ = 1.0 for both), and the T2D risk allele G was associated with higher *INHBB* expression levels in both muscle (*β* = 0.211, *p* = 2 × 10^−9^) and adipose (*β =* 0.252, *p* = 8 × 10^−42^). Notably, rs11688682 has no moderate LD proxies (*r*^2^ > 0.5 in EUR UKB), suggesting it may act as a causal variant. It is located approximately 244 kb downstream of the *INHBB* promoter in a region of open chromatin in muscle myoblasts and adipocytes, and it overlaps histone marks characteristic of enhancer elements^75^^,^^76^^,^^77^ (Figures 5A, 5B, and S9). *INHBB* is the closest gene and is highly expressed in adipose (average 94 TPM in bulk GTEx), moderately expressed in skeletal muscle and liver (6 TPM in both), and more lowly expressed in pancreas (2 TPM). These findings suggest *INHBB* as a strong candidate gene linking T2D genetic risk to regulatory mechanisms active in both muscle and adipose tissue.

We evaluated the regulatory function of rs11688682 using transcriptional reporter assays in four cell types: myoblasts and differentiated myocytes derived from the human skeletal LHCN-M2 cell line, and preadipocytes and differentiated adipocytes from the SGBS cell strain (Figures 5C, 5D, S10, and S11). In both cell lines, expression of *INHBB* increased by at least 7-fold after differentiation (Table S10). Across all four cell types, the T2D risk allele rs11688682^G^ showed higher transcriptional activity than the non-risk C allele, approximately 2.5-fold higher in myoblasts (*p* = 4.34 × 10^−7^) and 2-fold higher in adipocytes (*p* = 0.004). This direction of effect aligned with the eQTL results. Together, these findings suggest that the risk allele rs11688682^G^ increases enhancer activity, thereby upregulating *INHBB* expression in both tissues. This upregulation may influence activin and inhibin complex activity and ultimately contribute to increased T2D risk (Figure 5E). The hundreds of other colocalized GWAS-eQTL signals identified in this study offer a rich resource of putative regulatory variants and target genes for future functional validation (Table S7).

## Discussion

We conducted a large skeletal muscle eQTL study that identified 18,818 conditionally distinct eQTL signals across 12,283 eGenes, with 35% of eGenes harboring two or more signals. Compared to analyses of each contributing study individually, the meta-analysis identified over 20% more eGenes and more than 28% additional signals. One eGene highlighted for multiple signals that were only detectable in the meta-analysis, *SH3RF2*, is highly expressed in skeletal muscle, plays a role in skeletal muscle homeostasis during exercise, and has been broadly implicated in cellular apoptosis.^78^^,^^79^^,^^80^^,^^81^ Colocalization with GWAS data from 26 muscular and cardiometabolic traits revealed 2,252 GWAS-eQTL colocalizations, implicating 1,342 genes as potential contributors to trait or disease risk. Notably, only 37% of colocalizing GWAS signals were linked to their nearest protein-coding gene, suggesting that proximity alone is often insufficient for gene prioritization. 44% of GWAS-colocalized eGene TSSs were located more than 50 kb from the GWAS lead variant, underscoring the value of eQTL colocalization analyses for identifying regulatory mechanisms. One example colocalized gene, *PCK2*, was the fourth closest gene to the relevant T2D signal but is a compelling candidate gene for T2D, due to its role in skeletal muscle development and its involvement in glucose homeostasis through the stimulation of insulin release.^82^ By leveraging a larger sample size and conditional modeling, we enhanced the resolution and sensitivity of eGene and regulatory variant discovery, improving our ability to link GWAS signals to putative causal genes and regulatory elements in skeletal muscle.

We assessed colocalization with T2D signals and eQTLs from muscle and three additional trait-relevant tissues: adipose, liver, and pancreatic islets. We linked 309 T2D signals to 551 eGenes, including 95 eGenes identified exclusively in muscle. Cross-tissue colocalizations strengthened evidence for putative target genes. For example, a T2D signal with lead variant rs11688682 colocalized with eQTL signals for *INHBB* in both muscle and adipose tissue. This variant is located in regions of accessible chromatin in both cell types, and transcriptional reporter assays confirmed consistent regulatory activity across cell types, supporting the hypothesis that rs11688682 modulates expression of *INHBB* and contributes to increased T2D risk. Higher transcriptional activity with the rs11688682^G^ allele fragment in SGBS adipocytes compared to LHCN-M2 myoblasts may also suggest that cell type could impact transcription factor binding and gene expression. *INHBB* encodes a subunit of the dimeric activin and inhibin protein complexes and has been shown to influence skeletal muscle mass in mice.^62^^,^^63^ In human adipose tissue, it is downregulated during diet-induced weight loss and correlated with metabolic syndrome risk factors.^64^ Furthermore, *INHBB* has recently been approved as a drug target for pulmonary arterial hypertension.^83^ Many of the colocalized genes identified here may represent promising therapeutic targets. While incorporating multiple disease-relevant eQTL tissues improves gene prioritization, further increases in sample size and resolution will be critical to disentangle tissue-specific regulatory mechanisms from those limited by power constraints and allow for the identification of shared biological pathways or molecular functions driving these associations.

While we identified target genes for 309 T2D signals, relatively few were detected in more than one tissue. Although some may reflect tissue-specific regulatory effects, many single-tissue findings are likely attributable to limited ability to detect eQTLs in other tissues due to smaller eQTL sample size, low gene-expression levels, or variable data quality. For example, the adipose dataset, which had twice the sample size of the other tissues and identified twice as many eQTLs, yielded the largest number of T2D colocalizations. This suggests that many “adipose-only” colocalizations may also emerge in other tissues but remain undetected in current datasets. Despite the unbalanced sample sizes, integrating eQTL data across all four tissues in this study increased the number of colocalized T2D signals by 52% compared with adipose alone and by 255% compared with muscle alone. Other studies of T2D have also demonstrated the utility of analyzing eQTL datasets from multiple tissues. For example, a preprint^30^ reported colocalization of a similar set of T2D signals with eQTLs from seven GTEx tissues. Our pipeline and analysis of eQTLs from four tissues found a higher proportion of T2D signals colocalized with an eQTL (36% vs. 27%), highlighting the value of larger eQTL datasets and/or more rigorous signal isolation. These findings underscore the value of multi-tissue analyses while also highlighting the need for more deeply powered eQTL datasets to fully resolve tissue-specific gene regulation underlying complex traits such as T2D.

Nearly one in four colocalized eQTL signals were not primary signals, underscoring the importance of identifying conditionally distinct signals through stepwise analyses. These additional eQTLs can substantially improve the interpretation of GWAS associations by revealing regulatory mechanisms that may be overlooked when only primary signals are considered. In some cases, non-primary eQTLs colocalized with GWAS signals even when the corresponding primary eQTL did not. These may reflect signals with similar levels of association whose detection order differs between studies, potentially due to differences in allele frequency, effect size, or LD structure.^12^ Alternatively, they may represent regulatory effects active in distinct cell types within the bulk tissue, where only one cell-type-specific eQTL is relevant to the trait of interest.

While this skeletal muscle eQTL analysis substantially expands our understanding of gene regulation underlying complex traits, several limitations remain. First, although our study is relatively well powered for an eQTL, further increases in sample size would likely identify additional eGenes and regulatory signals not yet detected and reduce the potential for false-positive results. Expanding to include individuals from diverse ancestral backgrounds will be essential to identify population-dependent regulatory variants and improve the generalizability of findings. Second, our analyses identified more eQTL colocalizations using the European subset of the T2D GWAS compared with the multi-population T2D GWAS despite a smaller sample size, suggesting that our data and methods may be better suited to comparisons with European-stratified GWASs. Future work should develop and apply approaches optimized for cross-population integration to better leverage multi-population GWASs and improve gene discovery. Third, our meta-analysis was performed using bulk skeletal muscle tissue, which allows for the detection of many genes due to deep sequencing coverage but lacks resolution to distinguish cell-type-specific regulatory effects.^84^ Future studies could incorporate deconvolution with deeper sequencing data that represents all muscle tissue cells at higher resolution. Additionally, integrating chromatin accessibility data from specific muscle cell types could help prioritize regulatory variants acting in particular cellular contexts and clarify whether shared or distinct cell types mediate GWAS associations across tissues. Finally, while our reporter assays provide initial evidence that rs11688682 may have a disease-relevant regulatory effect on *INHBB*, additional experiments are needed to validate these findings. Additionally, future high-throughput methods will be valuable to further evaluate the putative target variants for these traits.

Altogether, this study generated a well-powered skeletal muscle eQTL resource, demonstrated the importance of incorporating eQTLs in multiple tissues to explain a heterogeneous disease, and expanded our knowledge of genes and regulatory variants that may influence muscular and cardiometabolic diseases. These data provide potential therapeutic targets and improve understanding of the genetic factors underlying complex traits and diseases.

## Data and code availability

The accession number for the data reported in this paper is Zenodo: 16647432. All results from this study are also available at https://mohlke.web.unc.edu/data/ and https://amp.colocus.app/.

## Acknowledgments

We acknowledge support from 10.13039/100000002National Institutes of Health grants R01 DK072193, UM1 DK126185, R01 DK093757, R01 DK062370, ZIA HG000024, F31 HL154730, and T32 HL129982. This research has been conducted with data from UK Biobank, a major biomedical database (http://www.ukbiobank.ac.uk/). The Genotype-Tissue Expression (GTEx) project was supported by the Common Fund of the Office of the Director of the 10.13039/100000002National Institutes of Health and by 10.13039/100000054NCI, 10.13039/100000051NHGRI, 10.13039/100000050NHLBI, 10.13039/100000026NIDA, 10.13039/100000025NIMH, and 10.13039/100000065NINDS. We thank the ENCODE Consortium for RNA-seq data. SGBS human preadipocytes were generously provided by Dr. Martin Wabitsch at the University of Ulm.

## Declaration of interests

The authors declare no competing interests.

## Web resources

APEX, https://github.com/lin-lab/apex
Apex2R, https://github.com/lin-lab/apex/tree/master/src/R
coloc v.5.1.0.1, https://chr1swallace.github.io/coloc/
GCTA, https://yanglab.westlake.edu.cn/software/gcta/#COJO
GENCODE v.19, https://www.gencodegenes.org/human/release_19.html#
GTEx lookup table and GENCODE v.26 gene annotation file, https://www.gtexportal.org/home/downloads/adult-gtex#reference
GTEx summary results, https://console.cloud.google.com/storage/browser/gtex-resources
METAL, https://github.com/statgen/METAL
Neale lab, GWAS summary statistics of UK Biobank, http://www.nealelab.is/uk-biobank/
PLINK v.1.90b3, https://www.cog-genomics.org/plink/
UCSC Genome Browser, https://genome.ucsc.edu/index.html
UK Biobank, http://www.ukbiobank.ac.uk/

## Supplemental information

Data S1. Tables S1–S10

Document S2. Article plus supplemental information
