# Supplementary material for: Skeletal muscle eQTL meta-analysis implicates genes in the genetic architecture of muscular and cardiometabolic traits
Source: Am J Hum Genet. Author manuscript; Available in PMC 2025 Nov 13. (PMC12614742; doi:10.1016/j.ajhg.2025.09.003)
Supplement: Supplementary file 1 [file NIHMS2112495-supplement-MMC1.pdf]

The American Journal of Human Genetics, Volume *112*

Supplemental information

Skeletal muscle eQTL meta-analysis implicates genes in the genetic architecture of muscular and cardiometabolic traits

Emma P. Wilson, K. Alaine Broadaway, Victoria A. Parsons, Swarooparani Vadlamudi, Narisu Narisu, Sarah M. Brotman, Kevin W. Currin, Heather M. Stringham, Michael R. Erdos, Ryan Welch, Jeffrey K. Holtzman, Timo A. Lakka, Markku Laakso, Jaakko Tuomilehto, Michael Boehnke, Heikki A. Koistinen, Francis S. Collins, Stephen C.J. Parker, Laura J. Scott, and Karen L. Mohlke

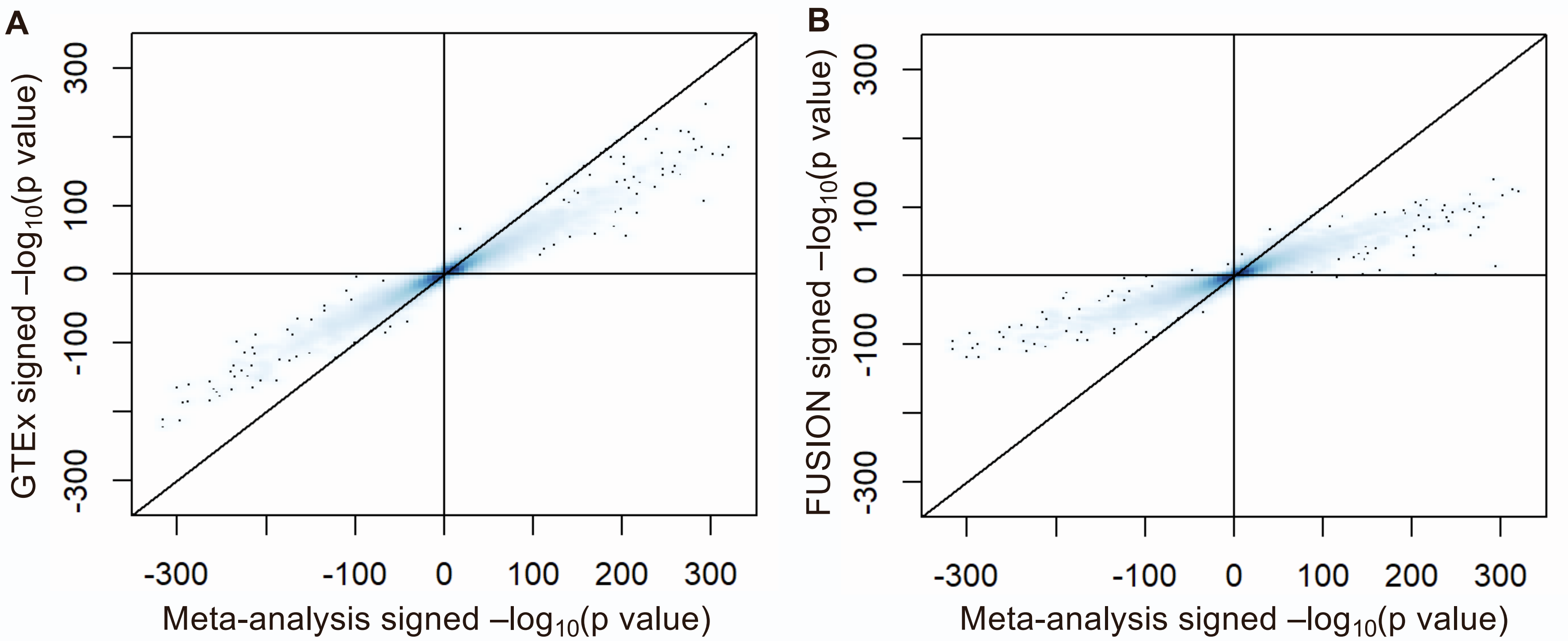

Figure S1. Comparisons of association strength between single studies and the meta-analysis. The *p* values of the lead variants for the 18,818 signals identified in the meta-analysis are compared between the meta-analysis and **(A)** GTEx alone or **(B)** FUSION alone.

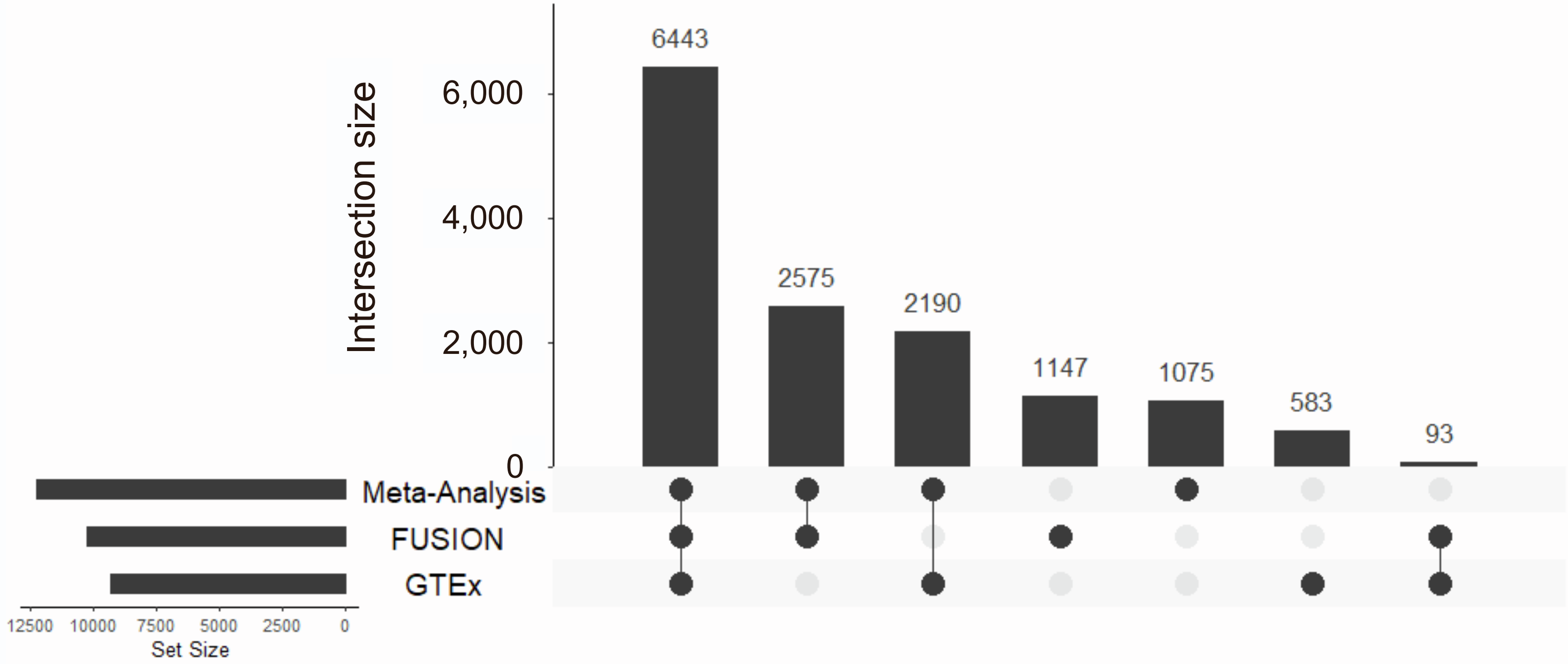

Figure S2. eGenes identified in the skeletal muscle eQTL meta-analysis and each individual study using APEX ($p < 1 \times 10^{-5}$). Of the 12,283 eGenes identified in the meta-analysis, 6,443 were also identified in both individual studies, 4,765 were identified in only one of the two studies, and 1,075 required the full power of the meta-analysis to be detected. 1,823 eGenes were detected in the single studies but not in the meta-analysis; these may correspond to true associations for transcripts that were not tested in the other study, variants that were excluded in the meta-analysis due to missingness in the other study, or effects that were heterogeneous between studies, or they may be false positives.

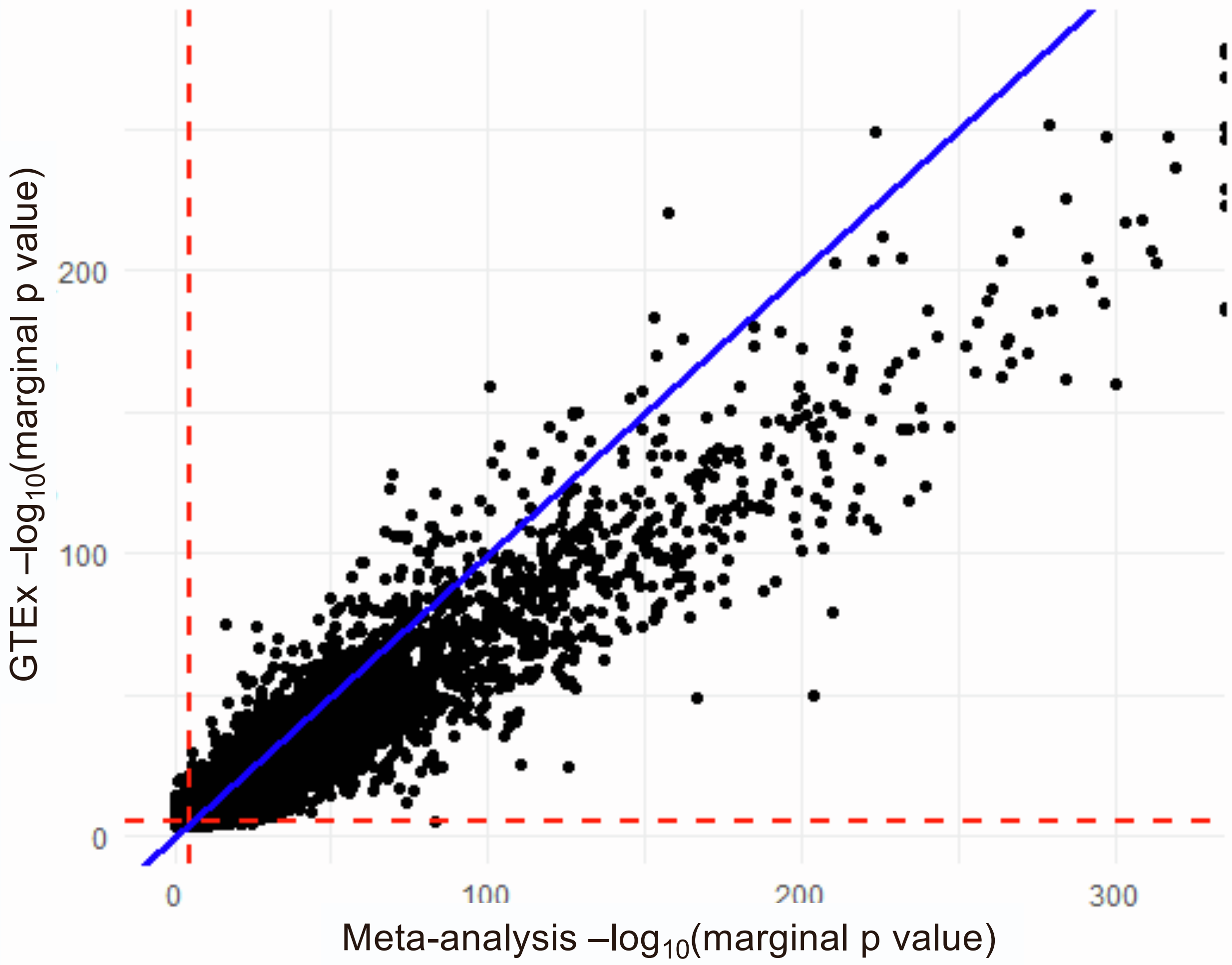

Figure S3. Lead variants of GTEx v8 skeletal muscle eGenes. $-\log_{10}$ marginal *p* values of 11,666 GTEx lead variants that were tested in the meta-analysis. Many variants showed stronger associations in the meta-analysis. Our significance threshold of 1×10^{-5} (red dashed lines) was more stringent than the GTEx threshold of FDR 5%. Blue line shows reference at y=x.

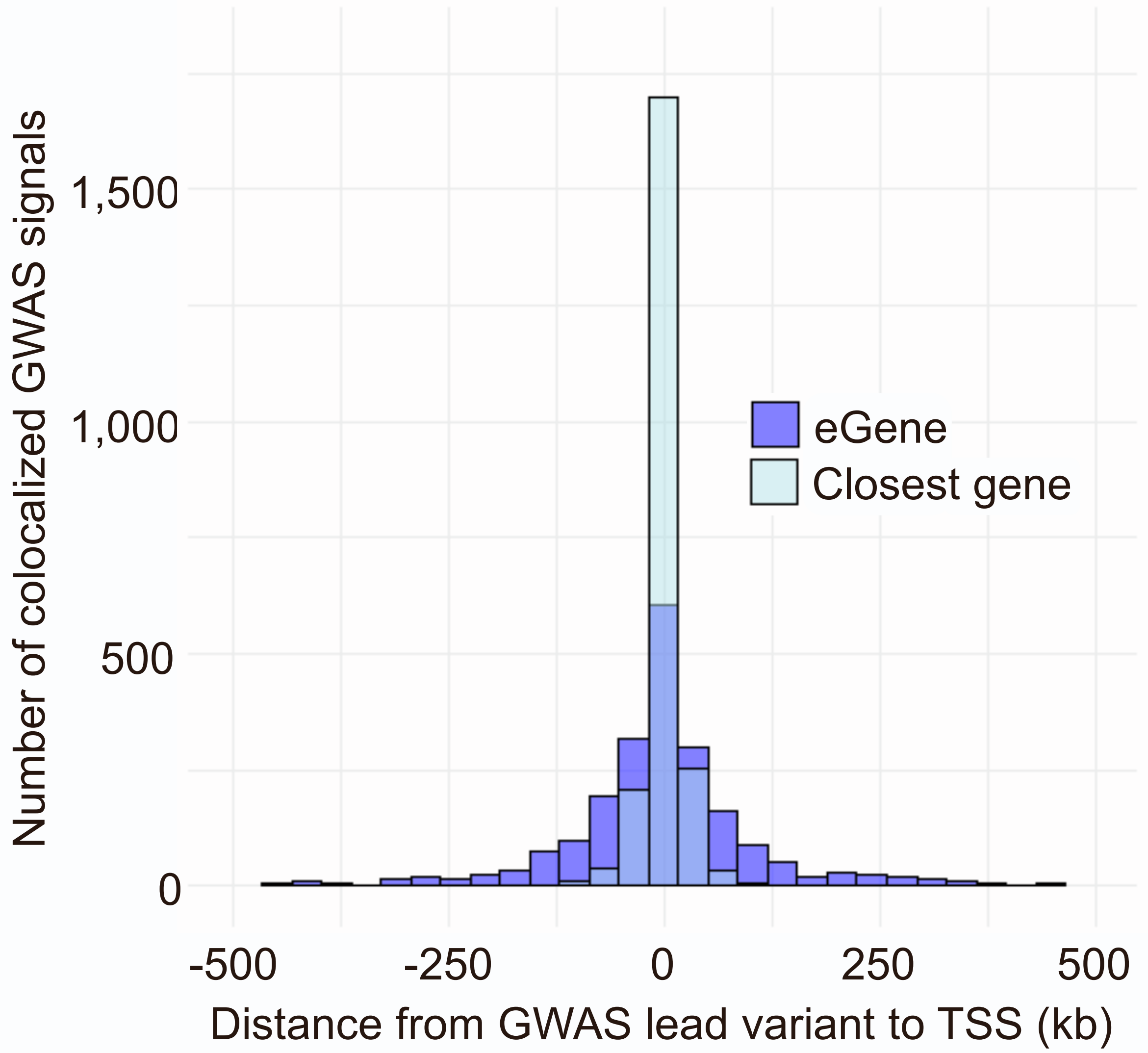

Figure S4. GWAS signals did not always colocalize with the nearest gene [all biotypes]. For the 2,252 colocalizations with an eQTL of any biotype, bars show the distance from the GWAS lead variant to the TSS of its nearest gene and the colocalized eGene. Plot is truncated at +/-500 kb.

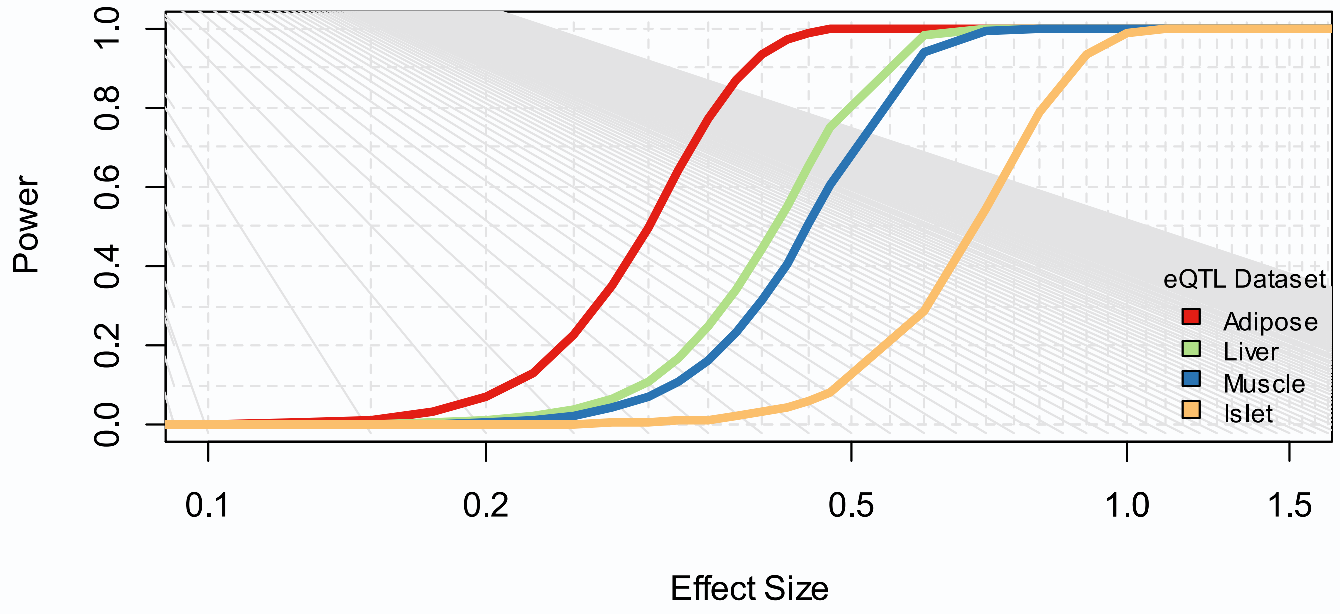

Figure S5. **Power estimation for eQTL studies tested for T2D colocalizations.** Power to detect eQTL for a variant at MAF=0.05 in sample sizes matching the eQTL data from adipose (n=2,256), liver (1,183), muscle (n=1,002), and islet (420). While sample size is a major determinant of power to detect eQTL, other potential factors include data quality, data source, and analysis pipeline.

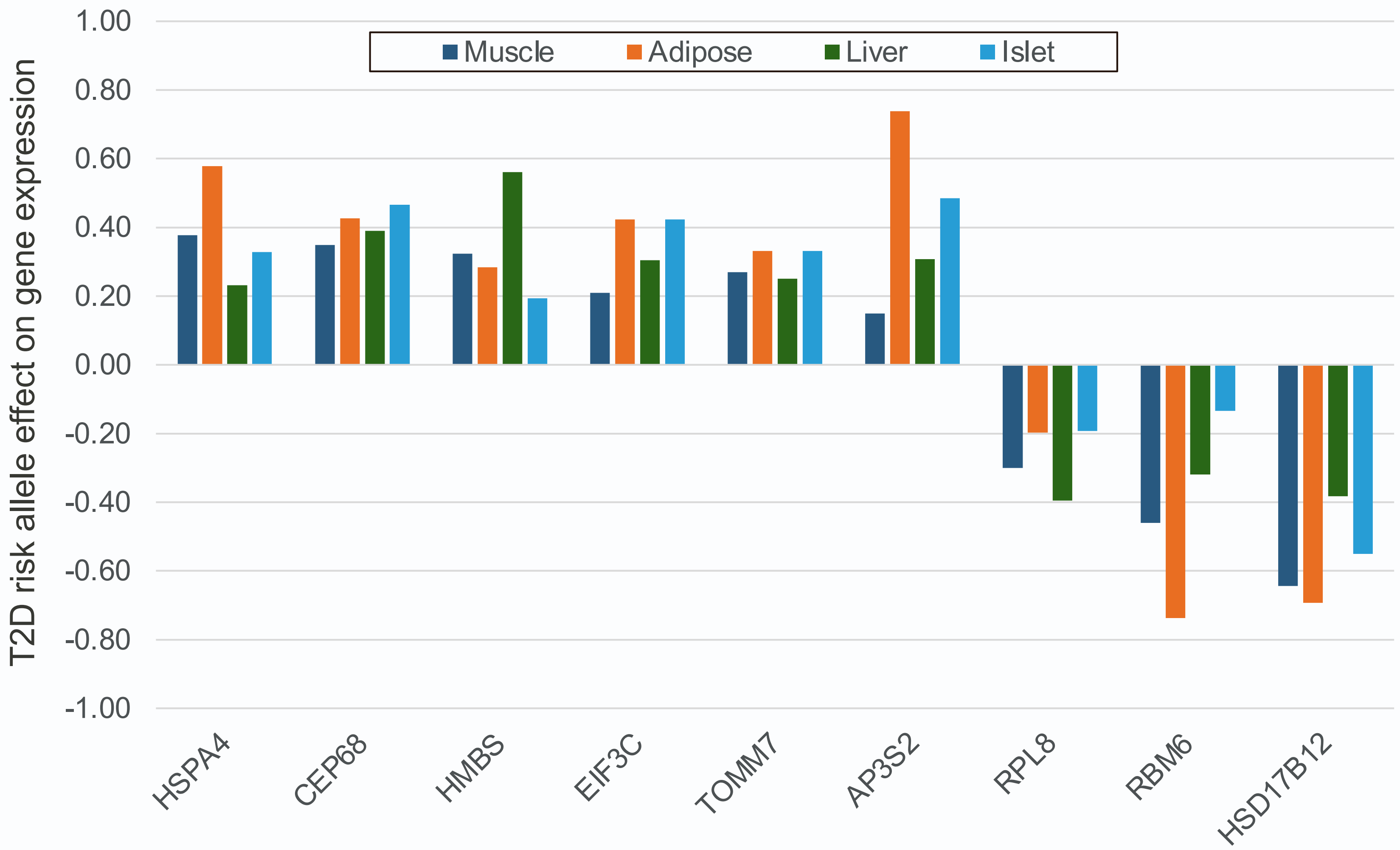

Figure S6. eQTL signals for nine genes colocalized with T2D in all four tissues. For each of the nine eGenes that had an eQTL signal colocalize with a T2D signal in all four tissues, bars show the effect size and direction of the T2D risk allele association with expression level.

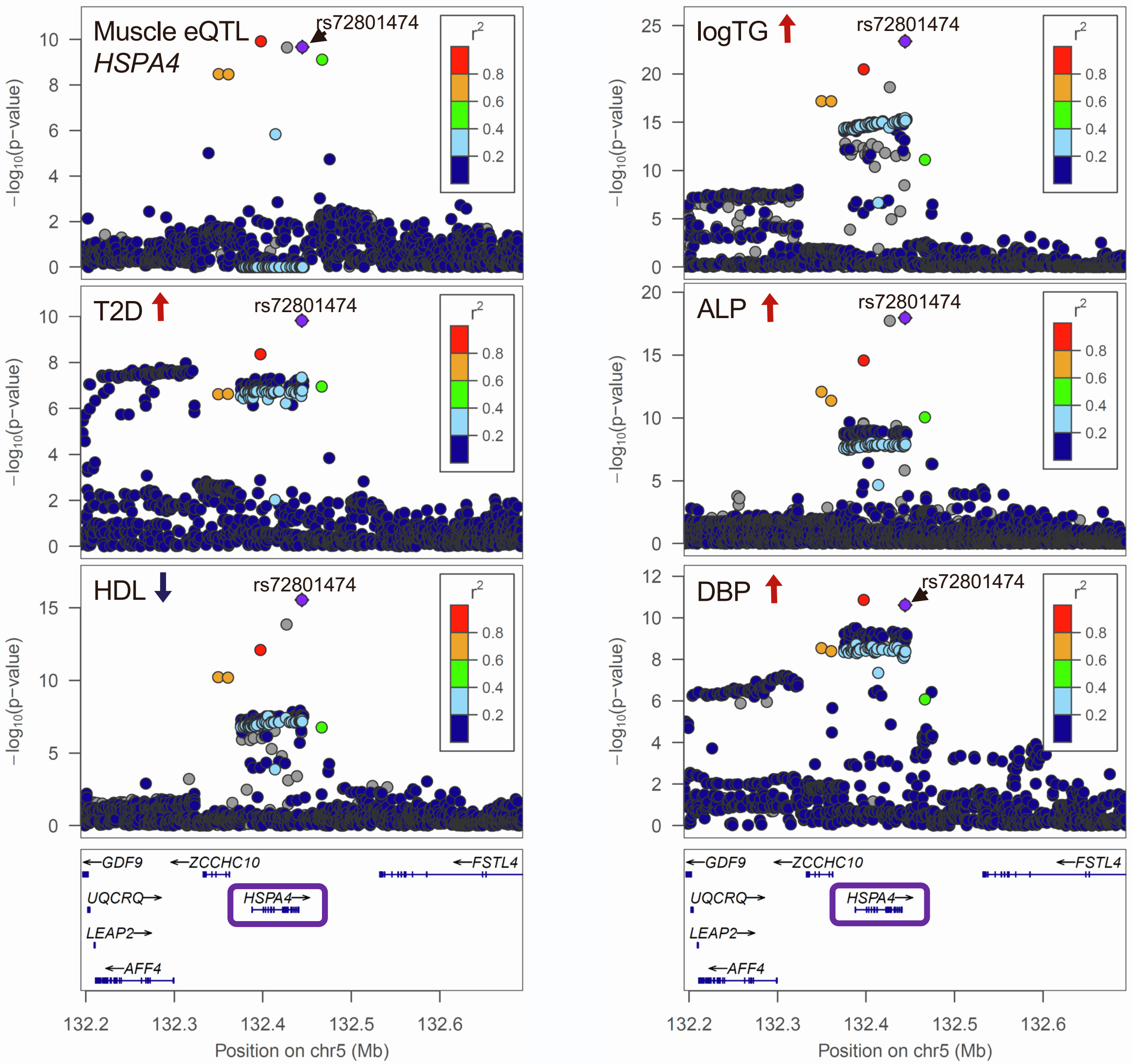

Figure S7. Muscle eQTL for *HSPA4* colocalized with T2D and additional cardiometabolic traits. A muscle eQTL signal for *HSPA4* colocalized with GWAS signals for T2D, HDL, logTG, ALP, and DBP (PP_{H4} > 0.99). Plots are colored by LD with T2D lead variant rs72801474. Arrows indicate the direction of effect of the expression-increasing allele with the trait.

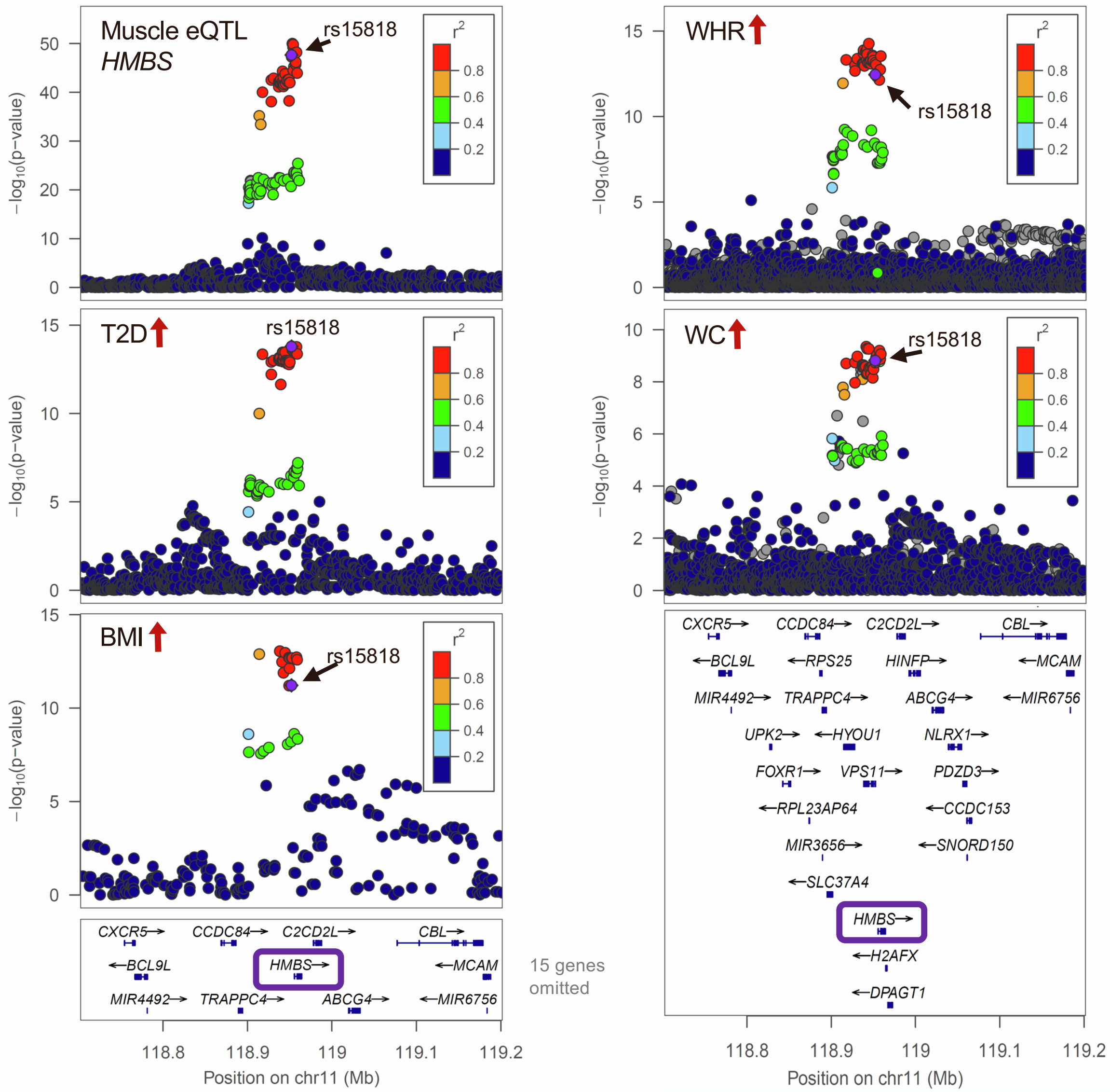

Figure S8. Muscle eQTL for *HMBS* colocalized with T2D and additional cardiometabolic traits. A muscle eQTL signal for *HMBS* colocalized with T2D, BMI, WHR, and WC ($PP_{H4} > 0.89$). Plots are colored by LD with T2D lead variant rs15818. Arrows indicate the direction of effect of the expression-increasing allele with the trait.

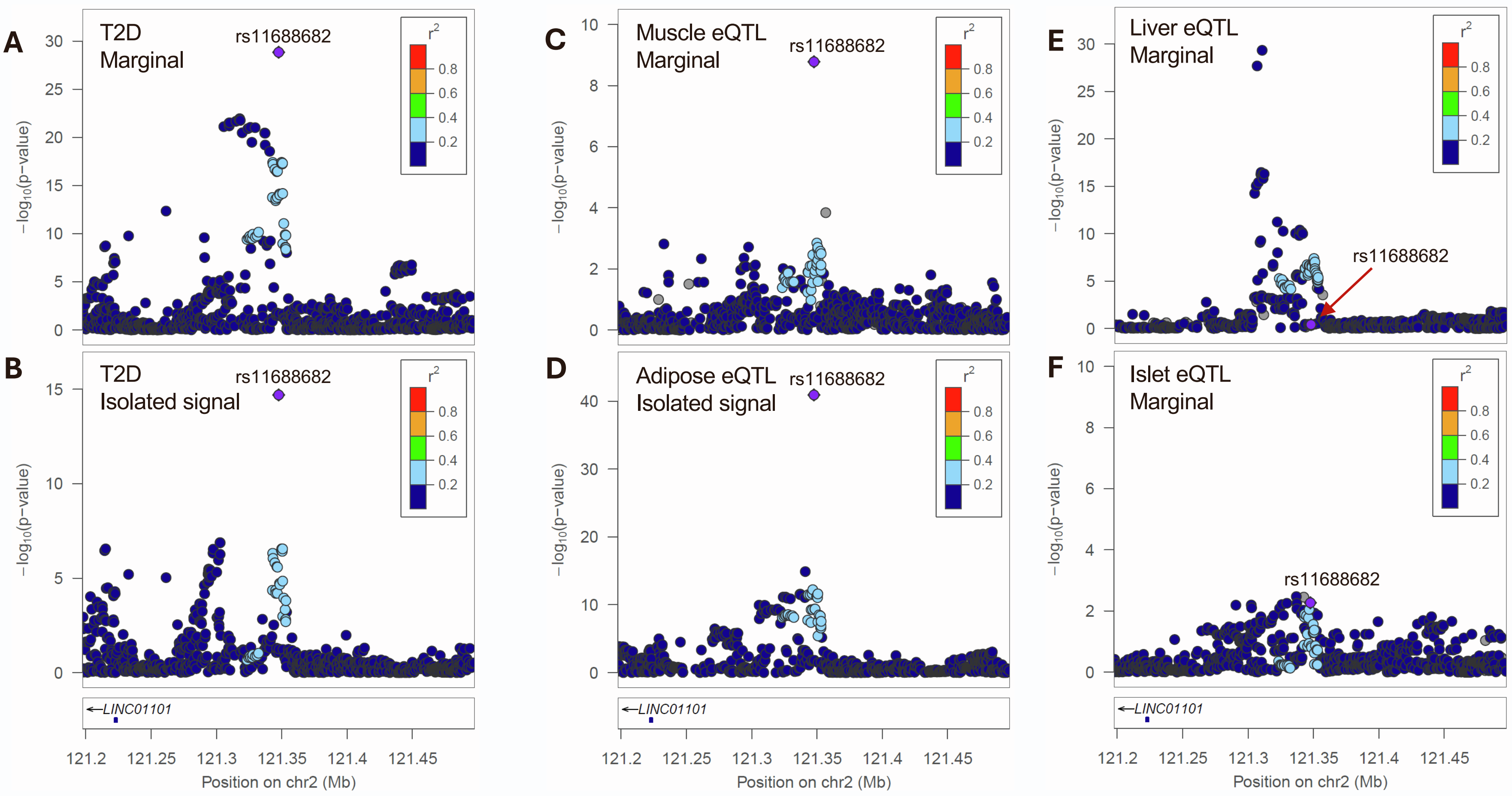

Figure S9. ***Colocalization between a T2D signal and an eQTL for INHBB in both muscle and adipose***. *INHBB* (not shown) is located ~244 kb upstream of rs11688682. **(A)** T2D marginal association data shows two signals in this region. **(B)** The primary T2D signal isolated for colocalization by conditional analysis. **(C)** A muscle eQTL for *INHBB* colocalized with the T2D signal and has the same lead variant (PP_{H4}=1). **(D)** The isolated primary *INHBB* eQTL signal in adipose colocalized with the T2D signal and has the same lead variant (PP_{H4}=1). **E)** Liver eQTL for *INHBB* detected a different signal from the signal at rs11688682. **F)** *INHBB* does not have a significant eQTL in pancreatic islets.

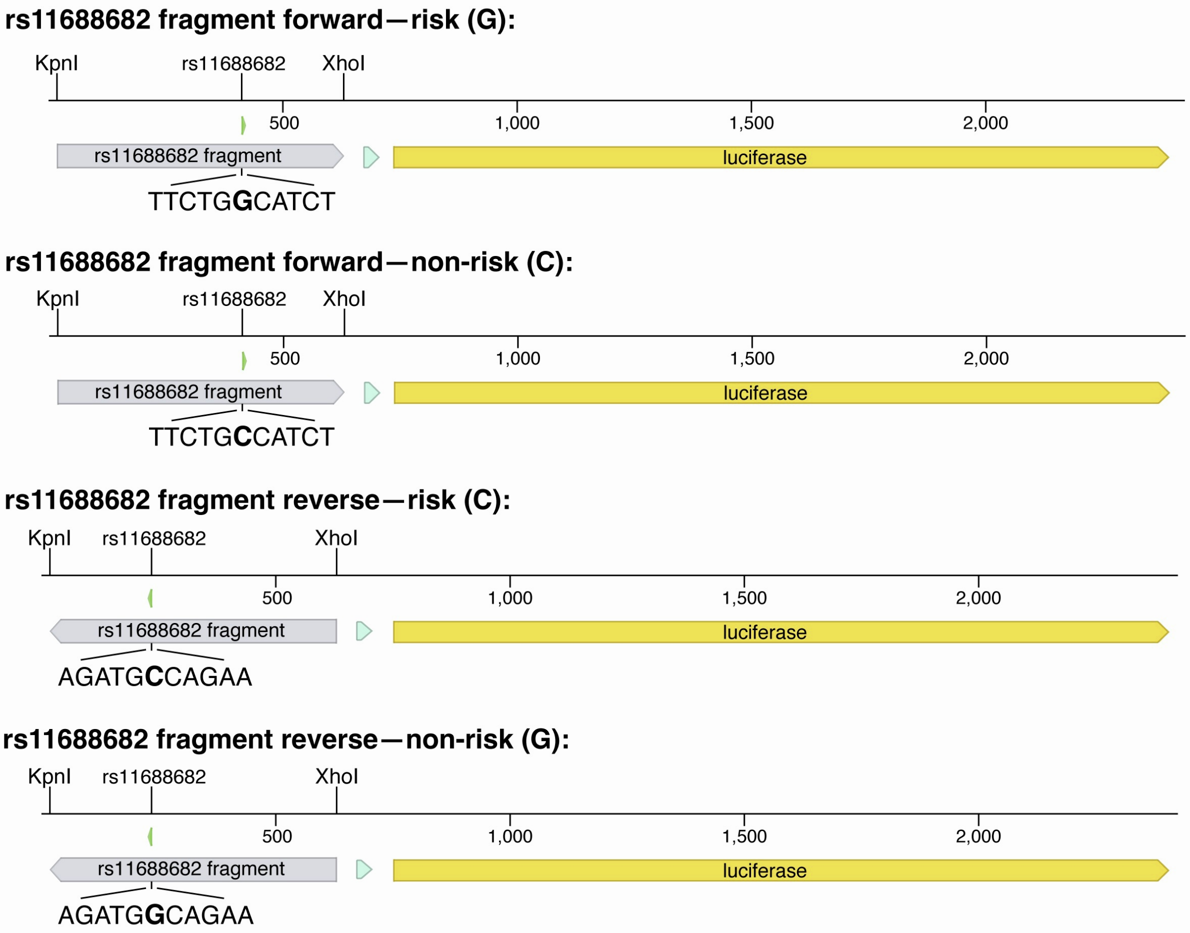

Figure S10. Diagrams of rs11688682 genomic fragments relative to luciferase gene in pGL4.23 plasmid. A 610-basepair fragment from chromosome 2 containing rs11688682 was ligated in either the forward or reverse orientation with respect to the genome upstream of the luciferase gene in the pGL4.23 plasmid. For each orientation (forward, reverse), fragments containing rs11688682–risk (G) and –non-risk (C) alleles were independently cloned, isolated, and verified by Sanger sequencing. Partial plasmid maps show the relative locations of the genomic fragment (gray), rs11688682 (green) with immediate surrounding sequence (below), minimal promoter (blue), and luciferase gene (yellow).

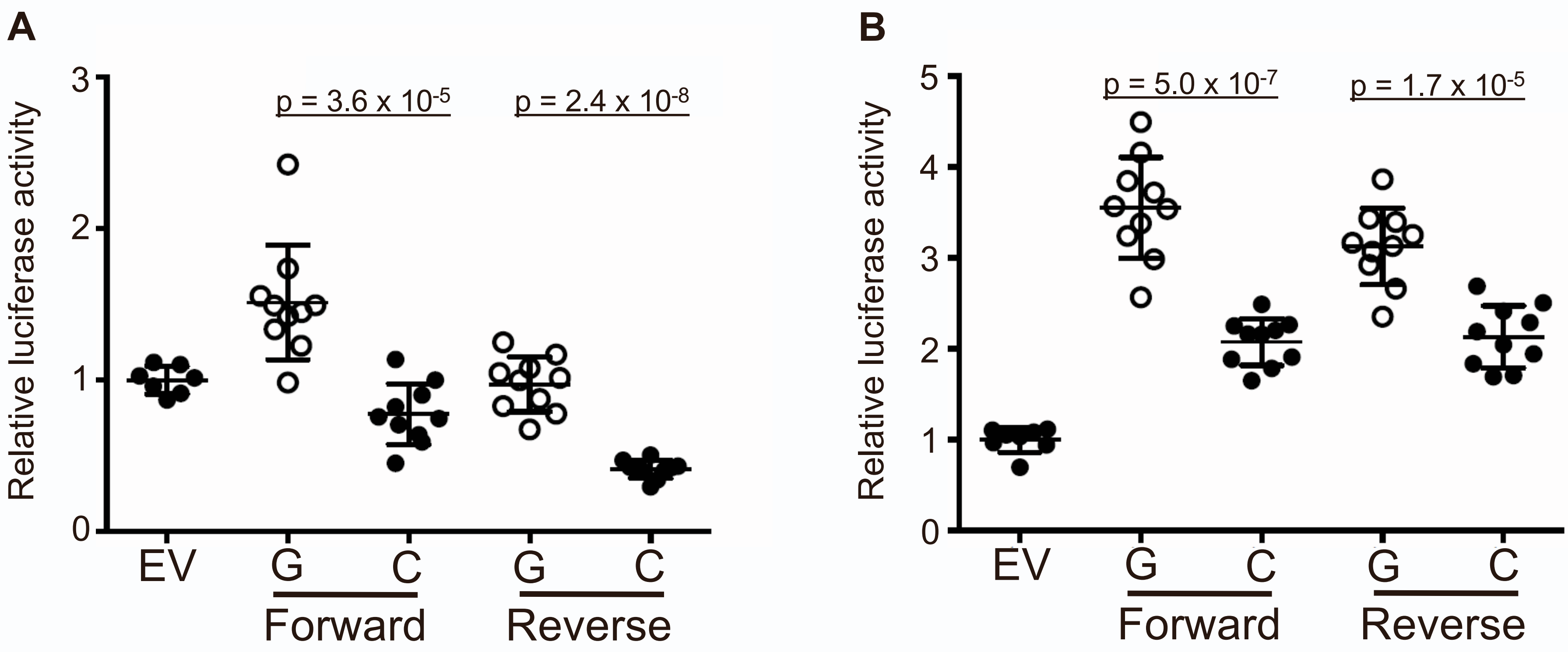

Figure S11. Transcriptional reporter assay results at rs11688682 in additional cell types. Luciferase activity of a 610-basepair fragment surrounding rs11688682. The fragment showed enhancer activity in transcriptional reporter assays in **(A)** LHCN-M2 differentiated myocytes and **(B)** SGBS-derived preadipocytes. The 7 to 10 points per allele or EV represent independent transfections. Bars show standard deviations; p-values correspond to two-sided t-tests.
